# Effectiveness of Robot-Assisted Upper Limb Training on Spasticity, Function and Muscle Activity in Chronic Stroke Patients Treated With Botulinum Toxin: A Randomized Single-Blinded Controlled Trial

**DOI:** 10.3389/fneur.2019.00041

**Published:** 2019-01-31

**Authors:** Marialuisa Gandolfi, Nicola Valè, Eleonora Kirilova Dimitrova, Stefano Mazzoleni, Elena Battini, Mirko Filippetti, Alessandro Picelli, Andrea Santamato, Michele Gravina, Leopold Saltuari, Nicola Smania

**Affiliations:** ^1^Department of Neurosciences, Biomedicine and Movement Sciences, University of Verona, Verona, Italy; ^2^UOC Neurorehabilitation, AOUI Verona, Verona, Italy; ^3^Polo Sant' Anna Valdera, Scuola Superiore Sant' Anna, The BioRobotics Institute, Pontedera, Italy; ^4^Physical Medicine and Rehabilitation Section, OORR Hospital, University of Foggia, Foggia, Italy; ^5^Research Department for Neurorehabilitation South Tyrol, Bolzano, Italy; ^6^Department of Neurology, Hochzirl Hospital, Zirl, Austria

**Keywords:** upper limb, rehabilitation, robotics, electromyography, spasticity

## Abstract

**Background:** The combined use of Robot-assisted UL training and Botulinum toxin (BoNT) appear to be a promising therapeutic synergism to improve UL function in chronic stroke patients.

**Objective:** To evaluate the effects of Robot-assisted UL training on UL spasticity, function, muscle strength and the electromyographic UL muscles activity in chronic stroke patients treated with Botulinum toxin.

**Methods:** This single-blind, randomized, controlled trial involved 32 chronic stroke outpatients with UL spastic hemiparesis. The experimental group (*n* = 16) received robot-assisted UL training and BoNT treatment. The control group (*n* = 16) received conventional treatment combined with BoNT treatment. Training protocols lasted for 5 weeks (45 min/session, two sessions/week). Before and after rehabilitation, a blinded rater evaluated patients. The primary outcome was the Modified Ashworth Scale (MAS). Secondary outcomes were the Fugl-Meyer Assessment Scale (FMA) and the Medical Research Council Scale (MRC). The electromyographic activity of 5 UL muscles during the “hand-to-mouth” task was explored only in the experimental group and 14 healthy age-matched controls using a surface Electromyography (EMGs).

**Results:** No significant between-group differences on the MAS and FMA were measured. The experimental group reported significantly greater improvements on UL muscle strength (*p* = 0.004; Cohen's d = 0.49), shoulder abduction (*p* = 0.039; Cohen's d = 0.42), external rotation (*p* = 0.019; Cohen's d = 0.72), and elbow flexion (*p* = 0.043; Cohen's d = 1.15) than the control group. Preliminary observation of muscular activity showed a different enhancement of the biceps brachii activation after the robot-assisted training.

**Conclusions:** Robot-assisted training is as effective as conventional training on muscle tone reduction when combined with Botulinum toxin in chronic stroke patients with UL spasticity. However, only the robot-assisted UL training contributed to improving muscle strength. The single-group analysis and the qualitative inspection of sEMG data performed in the experimental group showed improvement in the agonist muscles activity during the hand-to-mouth task.

**Clinical Trial Registration:** www.ClinicalTrials.gov, identifier: NCT03590314

## Introduction

Upper limb (UL) sensorimotor impairments are one of the major determinants of long-term disability in stroke survivors (1). Several disturbances are the manifestation of UL impairments after stroke (i.e., muscle weakness, changes in muscle tone, joint disturbances, impaired motor control). However, spasticity and weakness are the primary reason for rehabilitative intervention in the chronic stages (1–3). Historically, spasticity refers to a velocity-dependent increase in tonic stretch reflexes with exaggerated tendon jerks resulting from hyperexcitability of the stretch reflex (4) while weakness is the loss of the ability to generate the normal amount of force.

From 7 to 38% of post-stroke patients complain of UL spasticity in the first year (5). The pathophysiology of spasticity is complicated, and new knowledge has progressively challenged this definition. Processes involving central and peripheral mechanisms contribute to the spastic movement disorder resulting in abnormal regulation of tonic stretch reflex and increased muscle resistance of the passively stretched muscle and deficits in agonist and antagonist coactivation (6, 7). The resulting immobilization of the muscle at a fixed length for a prolonged time induces secondary biomechanical and viscoelastic properties changes in muscles and soft tissues, and pain (8–11). These peripheral mechanisms, in turn, leads to further stiffness, and viscoelastic muscle changes (2, 8). Whether the muscular properties changes may be adaptive and secondary to paresis are uncertain. However, the management of UL spasticity should combine treatment of both the neurogenic and peripheral components of spasticity (9, 10).

UL weakness after stroke is prevalent in both acute and chronic phases of recovery (3). It is a determinant of UL function in ADLs and other negative consequences such as bone mineral content (3), atrophy and altered muscle pattern of activation. Literature supports UL strengthening training effectiveness for all levels of impairment and in all stages of recovery (3). However, a small number of trials have been performed in chronic subgroup patients, and there is still controversy in including this procedure in UL rehabilitation (3).

Botulinum toxin (BoNT) injection in carefully selected muscles is a valuable treatment for spastic muscles in stroke patients improving deficits in agonist and antagonist coactivation, facilitating agonist recruitment and increasing active range of motion (6–8, 12–14). However, improvements in UL activity or performance is modest (13). With a view of improving UL function after stroke, moderate to high-quality evidence support combining BoNT treatment with other rehabilitation procedures (1, 9, 15). Specifically, the integration of robotics in the UL rehabilitation holds promise for developing high-intensity, repetitive, task-specific, interactive treatment of upper limb (15). The combined use of these procedures to compensate for their limitations has been studied in only one pilot RCT reporting positive results in UL function (Fugl-Meyer UL Assessment scale) and muscular activation pattern (16). With the limits of the small sample, the results support the value of combining high-intensity UL training by robotics and BoNT treatment in patients with UL spastic paresis.

Clinical scales are currently used to assess the rehabilitation treatment effects, but these outcome measures may suffer from some drawbacks that can be overcome by instrumental assessment as subjectivity, limited sensitivity, and the lack of information on the underlying training effects on motor control (17). Instrumental assessment, such as surface electromyography (sEMG) during a functional task execution allows assessing abnormal activation of spastic muscles and deficits of voluntary movements in patients with stroke.

Moreover, the hand-to-mouth task is representative of Activities of Daily Life (ADL) such as eating and drinking. Kinematic analysis of the hand-to-mouth task has been widely used to assess UL functions in individuals affected by neurological diseases showing adequate to more than adequate test-retest reliability in healthy subjects (18, 19). The task involves flexing the elbow a slightly flexing the shoulder against gravity, and it is considered to be a paradigmatic functional task for the assessment of spasticity and strength deficits on the elbow muscles (17, 20). Although sEMG has been reported to be a useful assessment procedure to detect muscle activity improvement after rehabilitation, limited results have been reported (16, 21).

The primary aim of this study was to explore the therapeutic synergisms of combined robot-assisted upper limb training and BoNT treatment on upper limb spasticity. The secondary aim was to evaluate the treatment effects on UL function, muscle strength, and the electromyographic activity of UL muscles during a functional task.

The combined treatment would contribute to decrease UL spasticity and improve function through a combination of training effects between BoNT neurolysis and the robotic treatment. A reduction of muscle tone would parallel improvement in muscle strength ought to the high-intensity, repetitive and task-specific robotic training. Since spasticity is associated with abnormal activation of shortening muscles and deficits in voluntary movement of the UL, the sEMG assessment would target these impairments (2, 8–11, 15).

## Materials and Methods

### Trial Design

A single-blind RCT with two parallel group is reported. The primary endpoint was the changes in UL spasticity while the secondary endpoints were changes in UL function, muscle strength and the electromyographic activity of UL muscles during a functional task. The study was conducted according to the tenets of the Declaration of Helsinki, the guidelines for Good Clinical Practice, and the Consolidated Standards of Reporting Trials (CONSORT), approved by the local Ethics Committee “Nucleo ricerca clinica–Research and Biostatistic Support Unit” (prog n.2366), and registered at clinical trial (NCT03590314).

### Patients

Chronic post-stroke patients with upper-limb spasticity referred to the Neurorehabilitation Unit (AOUI Verona) and the Physical Medicine and Rehabilitation Section, “OORR” Hospital (University of Foggia) were assessed for eligibility.

Inclusion criteria were: age > 18 years, diagnosis of ischemic or hemorrhagic first-ever stroke as documented by a computerized tomography scan or magnetic resonance imaging, at least 6 months since stroke, Modified Ashworth Scale (MAS) score (shoulder and elbow) ≤ 3 and ≥1+ (22), BoNT injection within the previous 12 weeks of at least one of muscles of the affected upper limb, Mini-Mental State Examination (MMSE) score ≥24 (23) and Trunk Control Test score = 100/100 (24).

Exclusion criteria were: any rehabilitation intervention in the 3 months before recruitment, bilateral cerebrovascular lesion, severe neuropsychologic impairment (global aphasia, severe attention deficit or neglect), joint orthopedic disorders.

All participants were informed regarding the experimental nature of the study. Informed consent was obtained from all subjects. The local ethics committee approved the study.

### Interventions

Each patient underwent a BoNT injection in the paretic limb. The dose of BoNT injected into the target muscle was based on the severity of spasticity in each case. Different commercial formulations of BoNT were used according to the pharmaceutical portfolio contracts of our Hospitals (Onabotulinumtoxin A, Abobotulinumtoxin A, and Incobotulinumtoxin A). The dose, volume and number of injection sites were set accordingly. A Logiq ® Book XP portable ultrasound system (GE Healthcare; Chalfont St. Giles, UK) was used to inject BoNT into the target muscle.

Before the start of the study authors designed the experimental (EG) and the control group (CG) protocols. Two physiotherapists, one for each group, carried out the rehabilitation procedures. Patients of both groups received ten individual sessions (45 min/session, two sessions/week, five consecutive weeks). Treatments were performed in the rehabilitative gym of the G. B. Rossi University Hospital Neurological Rehabilitation Unit, or “OORR” Hospital.

#### Robot-Assisted UL Training

The Robot-assisted UL Training group was treated using the electromechanical device Armotion (Reha Technology, Olten, Switzerland). It is an end-effector device that allows goal-directed arm movements in a bi-dimensional space with visual feedback. It offers different training modalities such as passive, active, passive-active, perturbative, and assistive modes. The robot can move, drive or oppose the patient's movement and allows creating a personalized treatment, varying parameters such as some repetitions, execution speed, resistance degree of motion. The exercises available from the software are supported by games that facilitate the functional use of the paretic arm (25). The robot is equipped with a control system called “impedance control” that modulates the robot movements for adapting to the motor behavior of the patient's upper limb. The joints involved in the exercises were the shoulder and the elbow, is the wrist fixed to the device.

**Figure 1:**
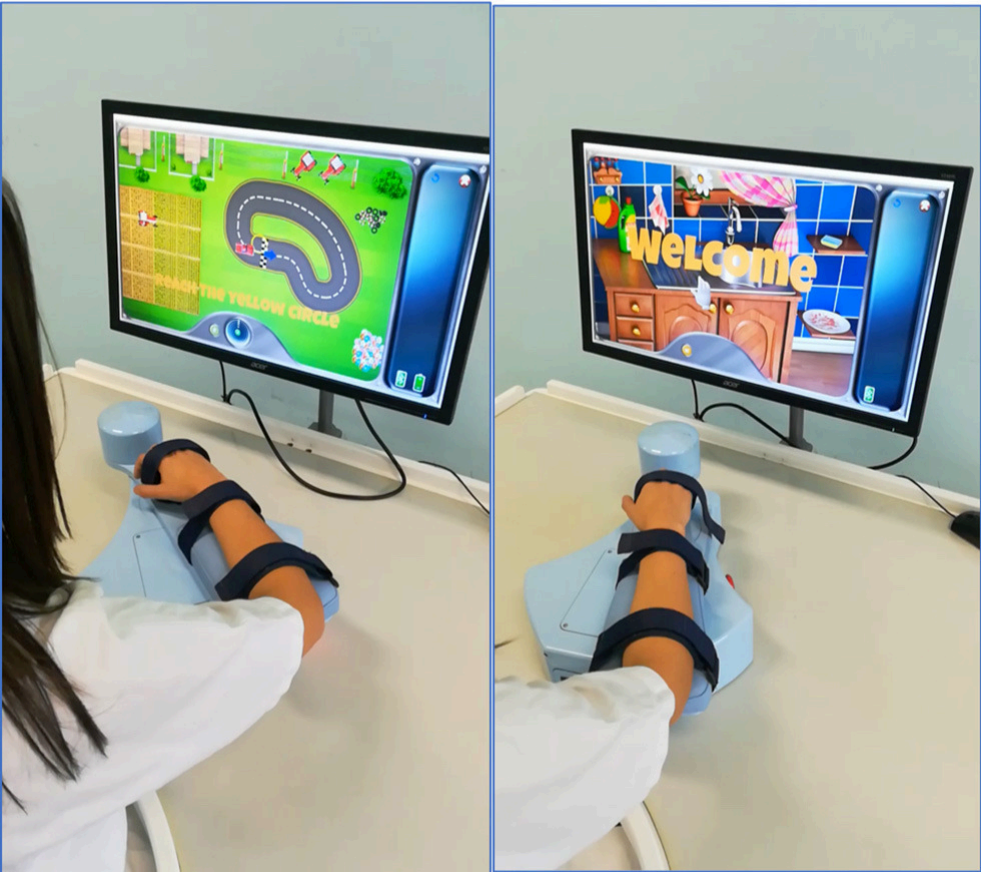
The upper limb robot-assisted training setting.

The Robot-assisted UL Training consisted of passive mobilization and stretching exercises for affected UL (10 min) followed by robot-assisted exercises (35 min). Four types of exercises contained within the Armotion software and amount of repetitions were selected as follows: (i) “Collect the coins” (45–75 coins/10 min), (ii) “Drive the car” (15–25 laps/10 min), (iii) “Wash the dishes” (40–60 repetitions/10 min), and (iv) “Burst the balloons” (100–150 balloons/5 min) (Figure 1). All exercises were oriented to achieving several goals in various directions, emphasizing the elbow flexion-extension and reaching movement. The robot allows participants to execute the exercises through an “assisted as needed” control strategy. For increment the difficulty, we have varied the assisted and non-assisted modality, increasing the number of repetitions over the study period.

#### Conventional Training

The conventional training consisted of UL passive mobilization and stretching (10 min) followed by UL exercises (35 min) that incorporated single or multi-joint movements for the scapula, shoulder, and elbow, performed in different positions (i.e., supine and standing position). The increase of difficulty and progression of intensity were obtained by increasing ROM, repetitions and performing movements against gravity or slight resistance (26). Training parameters were recorded on the patient's log.

#### Outcomes

Clinical and demographic data were collected at enrollment. The primary outcome was the changes on the UL MAS score computed as the sum of evaluation of shoulder, elbow, and wrist muscles (single-joint score range, 0–4; with higher scores indicating worse spasticity; total score, 0–12; with higher scores indicating worse spasticity) (16).

Secondary outcomes included changes on the Fugl Meyer Upper Limb Assessment scale (FMA) (27) (score range, 0–66; with higher scores indicating better performance), a widely used measure of UL function composed by 33 items assessing reflex activity, muscle strength, and movement control. Moreover, changes on the UL muscle strength were assessed by the Medical Research Council Scale (MRC) computed as the sum of the score from shoulder flexion/abduction/external rotation, elbow flexion/extension and wrist supination/flexion/extension (single-movement score range, 0–5; with higher scores indicating muscle strength against full resistance; total score, 0–40; with higher scores indicating muscle strength against full resistance) (28, 29).

Patients were assessed by a blinded rater before (T0) and post-treatment (T1). A subgroup of patients in the experimental group was investigated by surface Electromyography (EMGs) during the “hand-to-mouth” motor task (ARAT sub-item).

#### EMG Protocol

The subject seated in a comfortable position on a chair with a backrest, the feet resting on the floor and the knees and hips flexed at 90°. The start position consisted of the hand of the examined side lying on the distal third of the thigh. Then, the patient was asked to touch his mouth with the palm at average speed and return to the starting position. The patient was instructed not to move the head toward the hand. No other indications regarding how to move the arm for not to influence the spontaneity of the movement. The EMG activity of 5 upper limb muscles of the affected side (deltoid scapular, deltoid clavicular, pectoralis major–clavicular head, triceps brachii, biceps brachii) was measured using pairs of self-adhesive surface electrodes. Disposable Ag-AgCl electrodes were placed according to SENIAM guidelines with an inter-electrode spacing of 0.02 m. Before electrode placement, the skin was shaved with a disposable, single-use razor and cleaned with alcohol (30). Raw EMG signals were collected using BTS FREEEMG 300 wireless surface EMG sensors (BTS spa, Milan, Italy) at a sampling rate of 1,000 Hz. Raw EMG signals were processed with a customized routine developed in MATLAB environment (MathWorks, USA). The raw EMG signal was bandpass filtered at 20–450 Hz and then smoothed using a 20-ms root mean square (RMS) algorithm to obtain the envelope. Signals were recorded in three conditions: 30 s during resting position (basal), 5 s of maximal voluntary isometric contraction (MVIC), and during the hand-to-mouth task. The hand-to-mouth task has been widely used to assess UL functions in individuals affected by neurological diseases showing adequate to more than adequate test-retest reliability in healthy subjects (18, 19).

The task was divided into two sub-phases through the definition of three time-events: (1) start of the movement, (2) the moment when the hand touches the mouth, and (3) return to the initial position. The first sub-phase, named “elbow flexion phase,” was defined as the interval between the movement onset and the maximum elbow flexion. The second sub-phase, named “return phase,” refers to the interval between the maximum elbow flexion until movement offset after returning to the starting position. Normative data were collected from 14 healthy age-matched controls undergoing one EMGs acquisition. The time-events were determined using an accelerometer (BTS spa, Milan, Italy).

### Sample Size

Sample calculation took into account that in a similar study by Pennati et al. a difference in the MAS total score of 2.75 was detected between the experimental and the control group (pooled SD 2.77) because of the experimental rehabilitation (16). According to this study, a sample of at least 28 patients (14 per group) was estimated to have 80% power and an alpha (probability of type I error) of 5%. Assuming a 10% drop-out rate, 31 patients were necessary to perform the study.

### Randomization

The patients considered eligible were randomly assigned to the experimental group or control group (allocation ratio 1:1) by using a computer-generated random numbers list with simple randomization (www.randomization.org). Group allocation and the randomization list was kept concealed.

### Blinding

The same blinded examiner measured primary and secondary outcomes at each evaluation session. Another blinded assessor performed the EMG protocol.

### Statistical Analysis

An intention to treat analysis was used. Descriptive statistics included means, standard deviation and graph. The Shapiro-Wilk test was used to test data distribution. Parametric or non-parametric tests were used for inferential statistics, accordingly. The *T*-Test for unpaired data (or the Mann-Whitney test) was used for testing between-group differences at T0 and T1. For this purpose, we computed the changes of the score (Δ) between T0–T1. The T-Test for paired data (or Wilcoxon signed rank tests) was used to compare within-group changes over time. The effects size measures between the two independent groups (Cohen's d calculation) were used to evaluate the magnitude of the between-group treatment effects. The Pearson correlation test was performed for testing the association between FMA and MRC scores. The EMG signals were processed by using an adaptive pre-whitening filter and the approximated generalized likelihood-ratio (AGLR) algorithm to detect the muscle activity. The onset and offset of muscle activity were analyzed as the percentage of the movement cycle. A qualitative analysis of sEMG graphic records was carried out. Training efficiency was calculated as the number of repetitions divided by the number of minutes of active therapy (31). The level of significance was set *p* < 0.05. Software statistics SPSS 20.0 (IBM SPSS Statistics for Windows, Version 20.0, Armonk, NY, USA).

## Results

**Figure 2:**
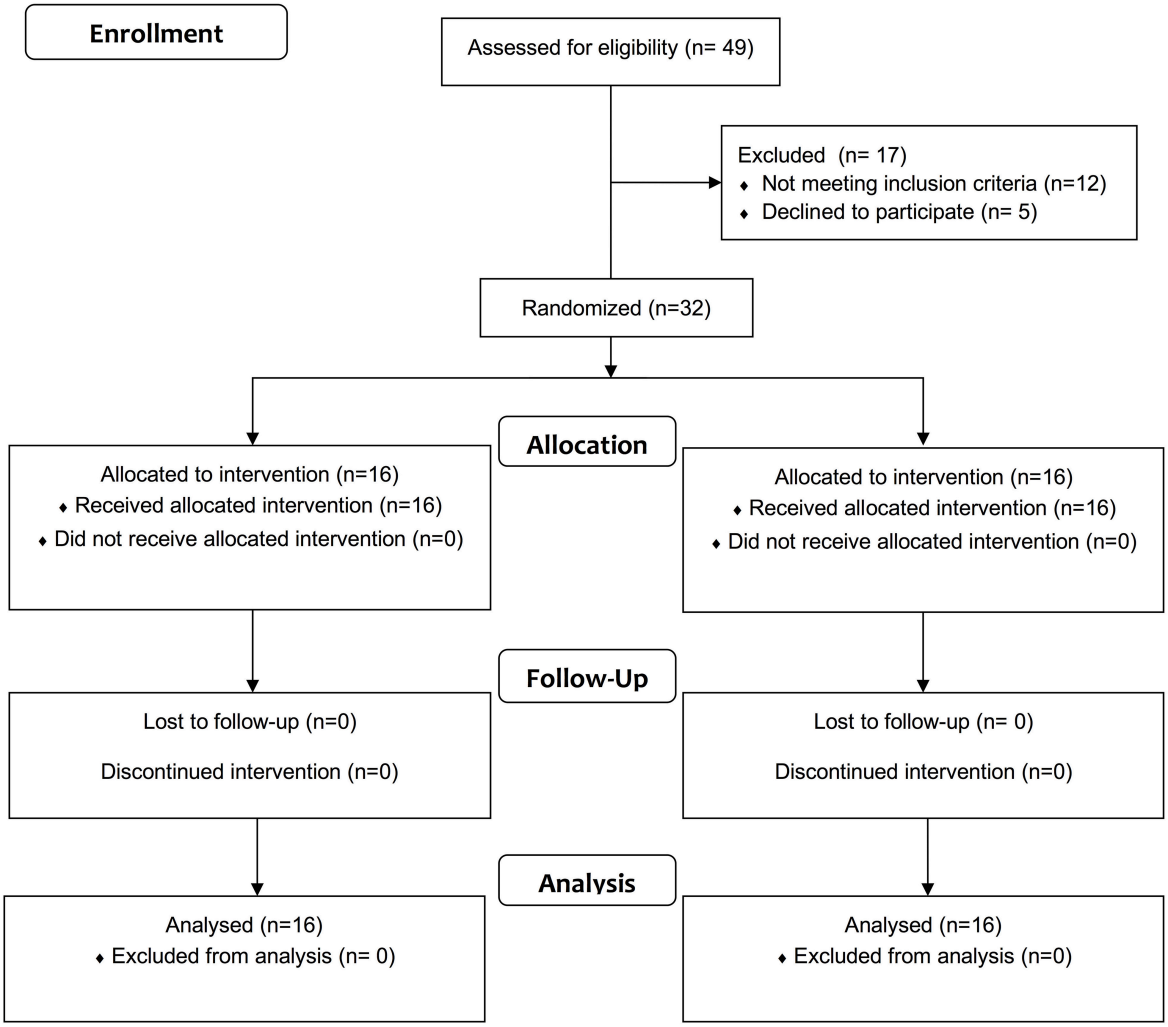
Flow diagram of the study.

Forty nine patients were evaluated for eligibility between February 2017 and April 2018. Twelve patients were excluded because they did not meet the inclusion criteria and five declined to participate. Thus, 32 patients were randomly allocated to the EG (*n* = 16) or CG (*n* = 16). All patients complete the study (Figure 2 and Table 1). A mean training efficiency of 7.3 repetitions/min and 2.2 repetitions/min was computed in the EG and CG, respectively. Matching in initial between-group conditions no significant differences as to age, disease duration, and all baseline clinical measures at T0 were measured (Tables 1, 2).

**Table 1:** Demographic and clinical characteristics of treated subjects.

	**Experimental group**	**Control group**	**Between-group comparison**
	**(*n =* 16)**	**(*n =* 16)**	
Age (years) Mean *(SD)* Range	59.31 (14.40) 21–77	59.13 (14.97) 21–78	n.s.
Gender (%) Male/Female	12/4	10/6	n.s.
Disease duration since diagnosis (years) Mean *(SD)*	6.0 (3.1)	5.1 (2.2)	n.s.
Side of paresis (%) Right/Left	62.5/37.5	50/50	n.s.
Modified Barthel Index Mean *(SD)* Range	68.75 (19.87) 35–95	68.13 (16) 35–95	n.s.

*SD, standard deviation; n.s, not significant*.

**Table 2:** Clinical outcome measures and inferential statistics.

				**Mean between-group differences**	**Between groups comparison**		**Mean within-group differences**	**Within-groups comparison**
		**T0**	**T1**	**T1**	**T0**	**T1–T0**	**T1–T0**	**T1–T0**
**Outcome Measure**	**Group**	**Mean** ***(SD)*** **Median [Q1; Q3]**	**Mean** ***(SD)*** **Median [Q1; Q3]**	**Mean (LB; UB) 95% CI**	***p***	**P (ES)**	**Mean (LB; UB) 95% CI**	***p***
**PRIMARY OUTCOME MEASURE**								
MAS^**γ**^ upper limb	EG	3.75 [3.00; 6.62]	3.50 [2.00; 4.75]	−0.12 (−1.48; 1.23)	n.s	n.s (−0.02)	−1.18 (−2.10; −0.27)	0.008
	CG	4.25 [3.25; 5.37]	3.00 [2.00; 5.50]				−1.15 (−1.72; −0.59)	0.003
**SECONDARY OUTCOME MEASURES**								
FMA	EG	28.75 (11.92)	32.38 (11.84)	2.12 (−6.81; 11.06)	n.s	n.s (−0.17)	3.62 (1.77; 5.48)	0.001
	CG	27.94 (10.82)	34.5 (12.89)				6.56 (3.75; 9.36)	0.001
**MRC**								
Total UL	EG	23.00 [14.37; 25.25]	24.75 [16.37; 27.37]	0.56 (−4.50; 5.63)	n.s	0.004 (0.49)	3.62 (2.16; 5.08)	0.001
	CG	23.00 [16.12; 28.37]	26.25 [17.75; 28.37]				0.90 (−0.31; 2.13)	n.s.
Shoulder flexion^**γ**^	EG	3.00 [2.00; 3.50]	3.00 [3.00; 4.00]	0.37 (−0.32; 1.07)	n.s	n.s.	0.40 (−0.14; 0.66)	0.01
	CG	3.00 [3.00; 4.00]	4.00 [3.50; 4.00]				0.34 (0.13; 0.55)	0.009
Shoulder abduction^**γ**^	EG	3.00 [2.00; 4.00]	3.25 [3.00; 4.00]	0.21 (−0.22; 0.65)	n.s	0.039 (−0.42)	0.62 (0.25; 0.99)	0.007
	CG	3.50 [3.00; 4.00]	3.50 [3.12; 4.00]				0.12 (−0.02; 0.27)	n.s.
Shoulder's external rotation^**γ**^	EG	2.00 [2.00; 3.00]	3.00 [2.00; 3.00]	0.00 (−0.80; 0.80)	n.s	0.019 (−0.72)	0.53 (0.26; 0.79)	0.004
	CG	3.00 [2.00; 3.00]	3.00 [3.00; 3.37]				−0.06 (−0.43; 0.31)	n.s.
Elbow flexion^**γ**^	EG	3.00 [2.00; 4.00]	4.00 [3.00; 4.00]	−0.25 (−0.77; 0.27)	n.s	0.043 (−1.15)	0.59 (0.16; 1.02)	0.008
	CG	3.00 [3.00; 3.50]	3.00 [3.00; 3.50]				0.00 (−0.25; 0.25)	n.s.
Elbow extension^**γ**^	EG	3.25 [2.00; 4.00]	4.00 [2.25; 4.00]	0.00 (−0.81; 0.81)	n.s	n.s.	0.53 (−0.11; 0.94)	0.017
	CG	3.50 [3.00; 4.00]	3.50 [3.12; 4.00]				0.25 (0.03; 0.46)	0.038
Forearm's supination^**γ**^	EG	2.50 [1.00; 3.00]	3.00 [1.00; 3.37]	0.25 (−0.89;1.39)	n.s	n.s.	0.40 (0.08; 0.73)	0.024
	CG	3.00 [0.25; 4.00]	3.75 [1.00; 4.00]				0.21 (−0.05; 0.49)	n.s.
Wrist flexion^**γ**^	EG	2.00 [0.25; 3.37]	3.00 [1;00; 3.87]	0.12 (−0.90; 1.15)	n.s	n.s.	0.31 (−0.01; 0.63)	n.s.
	CG	3.00 [1.00; 3.87]	3.00 [1.00; 3.87]				−0.03 (−0.33; 0.26)	n.s.
Wrist extension^**γ**^	EG	2.00 [0.25; 3.00]	2.00 [1.25; 4.00]	−0.15 (−1.20; 0.89)	n.s	n.s.	0.21 (−0.07; 0.51)	n.s.
	CG	2.50 [0.25; 3.37]	2.50 [1.00; 3.00]				0.06 (−0.33; 0.46)	n.s.

γ *non-parametric statistics*.

*P ≤ 0.05*.

*FMA, Fugl Meyer Assessment; MAS, Modified Ashworth Scale; MRC, Medical Research Council; ES. Effect size; EG, Experimental Group; CG, Control Group; CI, Confidence Interval; SD, Standard Deviation; Q1, 25° interquartile; Q3, 75° interquartile; LB, Lower Bound; UB, Upper Bound; n.s., not significant*.

### Primary Outcome Measure

Both groups reduced UL spasticity significantly without reporting significant between-group differences (Table 2).

### Secondary Outcome Measures

**Figure 3:**
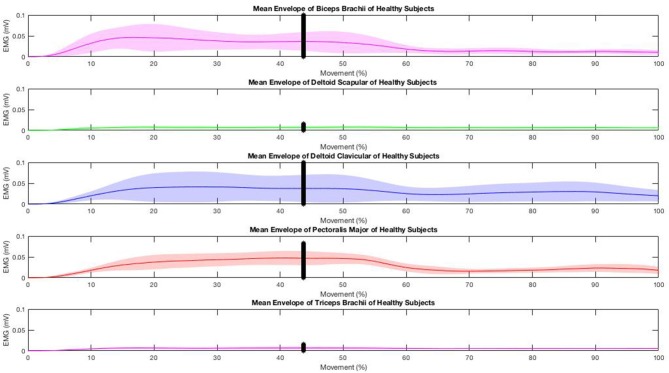
EMGs muscle activity during the “hand-to-mouth” task in healthy controls.

**Figure 4:**
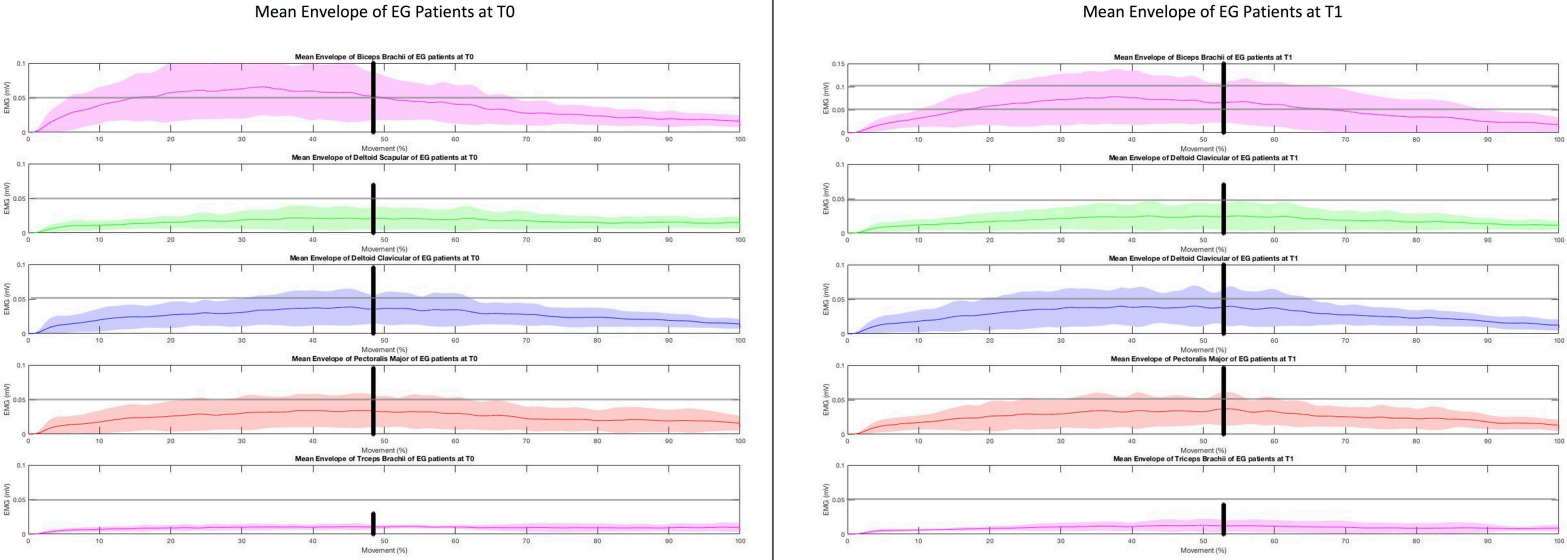
EMGs muscle activity during the “hand-to-mouth” task in stroke patients before and after the upper limb Robot-assisted training.

**Figure 5:**
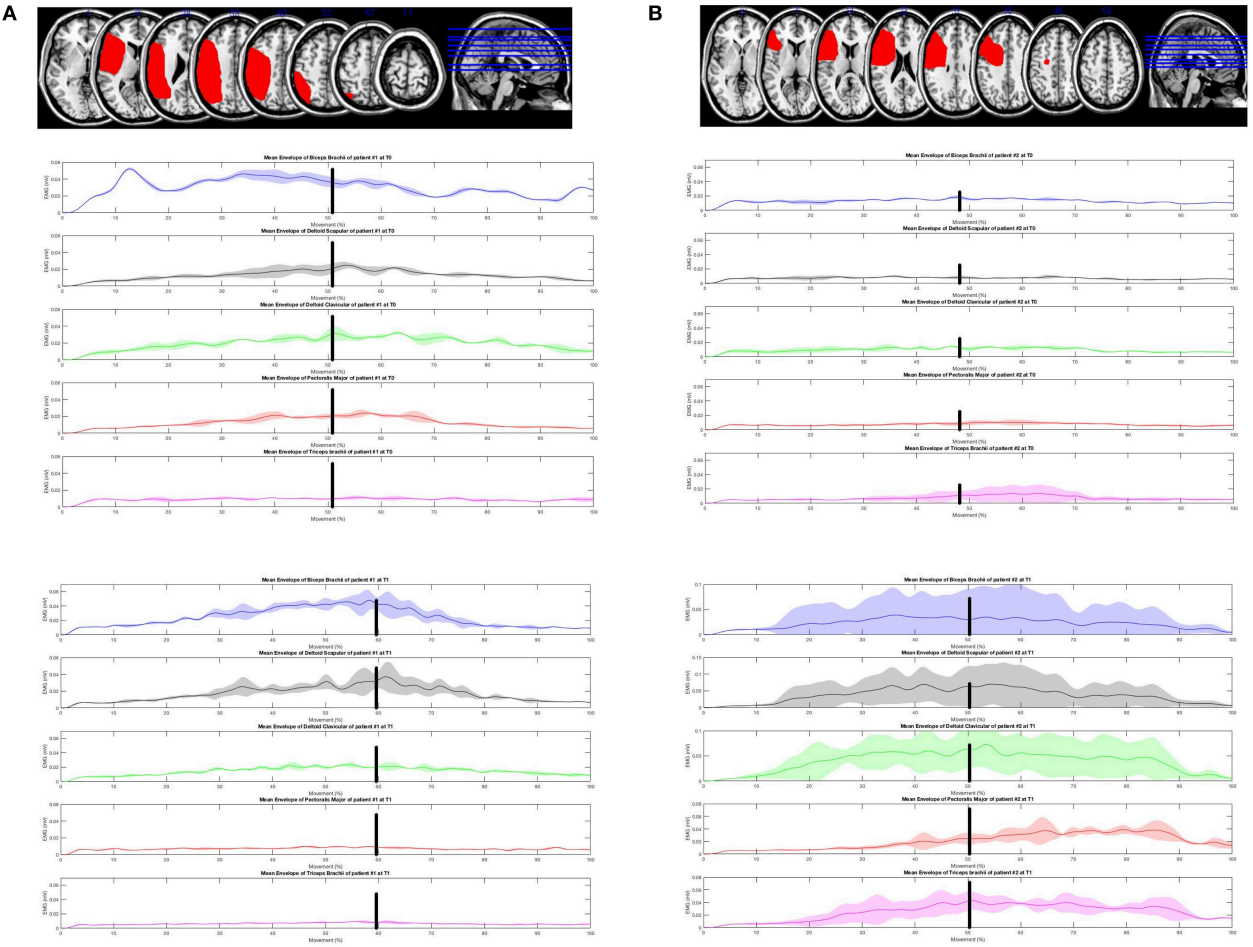
Lesions displayed on the magnetic resonance imaging brain template and electromyographic activity of the five upper limb muscles during the “hand-to-mouth” task was explored using a surface Electromyography (EMGs) in two patients. **(A)** Top left: Lesion Mapping (patient 1). Magnetic resonance imaging showed a lesion involving the left fronto-parietal lobes. Brodmann areas 48, 40, 39, 6, 44, 45, 3, 7, 22, 41, 2, 42, 4, 19, 43, 37, 21,47,9, 46, 1, 18, 23, 10 (MRIcron software, http://www.mricro.com/mricron). The electromyographic activity of the 5 UL muscles during the “hand-to-mouth” task was explored using a surface Electromyography (EMGs). **(B)** Top right: Lesion Mapping (patient 2). Magnetic resonance imaging showed a lesion involving the left frontal lobe. Brodmann areas 48, 45, 44, 6, 46, 43, 4, 3, 47, 32, 9, 38, 22, 10 (MRIcron software, http://www.mricro.com/mricron). The electromyographic activity of the 5 UL muscles during the “hand-to-mouth” task was explored using a surface Electromyography (EMGs).

Both groups improved UL function significantly without significant between-group differences (Table 2). The experimental group reported significantly greater improvements on UL muscle strength (delta T1–T0 = 3.62, *p* = 0.004, Cohen's d = −0.49), shoulder abduction (delta T1–T0 = 0.62, *p* = 0.039, Cohen's d = −0.42), external rotation (delta T1–T0 = 0.53, *p* = 0.019, Cohen's d = −0.72) and elbow flexion (delta T1–T0 = 0.59, *p* = 0.043, Cohen's d = −1.15) than the control group (Table 2). Changes in UL muscle strength were significantly correlated with changes in FMA (*r* = 0.49, *p* = 0.05). The results of sEMG analysis are reported in Figures 3–**5**. The mean envelopes of sEMG signals of the healthy subjects and patients of the EG are shown as a function of the movement progression.

Based on the results, the mean sEMG envelope computed on the healthy subjects' recordings (Figure 3) shows a low but constant recruitment of scapular deltoid and triceps brachii during the entire movement. On the other hand, the activation of the biceps brachii, clavicular deltoid, and pectoralis major is higher during the first phase and decreases during the second phase. It corresponds to the typical muscular recruitment occurring during the execution of the required reaching movement (32).

The same mean sEMG enveloped computed on stroke patients at T0 (Figure 4, left) showed a similar trend, especially in the first phase: during the second phase the decrease of the biceps brachii, clavicular deltoid and pectoralis major is higher than that observed in the healthy subjects.

At T1 (Figure 4, right) the time of sustained activation of the biceps brachii is much longer if compared to the activation observed at T0: in fact, the decrease which starts at about 70% of the movement corresponds to a double value if compared to T0 (~0.06 vs. 0.03 mV). In addition, the value observed in the healthy subjects at the transition of the two phases is ~0.035 mV, the corresponding value observed in the subjects at T1 is ~0.7 mV: this higher mean sEMG activation value corresponds to a different abnormal recruitment pattern of this muscle in the recruited patients.

As regards the analysis of the mean sEMG envelope of single subjects, patient #1 at T0 (Figure 5 top left) shows a poor modulation of recorded muscles which is more regular at T1 (Figure 5 bottom left). The activation of patient #2 at T0 is almost absent (Figure 5 top right), while at T1 (Figure 5 bottom right) the modulation of muscles is clear. In both cases the plots at T1 highlight a change of the muscular recruitment probably due to the proposed upper limb training program.

## Discussion

The main finding of this work is that robot-assisted training is as effective as conventional training on muscle tone reduction when combined with Botulinum toxin in chronic stroke patients with UL spasticity. However, only the robot-assisted UL training contributed to improving muscle strength with a moderate positive correlation with UL function. Preliminary observation of muscular activity in the experimental group showed, in some subjects, enhancement of the agonist muscles activity during the hand-to-mouth task. It might suggest a task-specific effect of the robot-assisted approach on muscle activity during the functional task. However, the sEMG protocol was not focused on investigating the relationship between abnormal activation of agonists and antagonists' muscles as it was limited to a qualitative analysis of sEMG envelope.

The combined use of BoNT injection and robot-assisted UL training appears to be a promising combination to target the different sensorimotor impairments because spasticity is associated with abnormal activation of shortening muscles and deficits in the UL voluntary movements (2, 8–11, 15). On the one hand, BoNT appears to be effective in the reduction of the neural components of the spastic movement disorders facilitating agonist recruitment and decreasing co-contraction of the antagonist's muscles. On the other hand, robotic devices can reduce muscle tone and motor impairment (1).

Given the multiplicity of symptoms that often need to be addressed in the UL rehabilitation of stroke patients, the integration of robotics holds promise for developing high-intensity, repetitive, task-specific, interactive treatment (15). Despite significant heterogeneity in the robotic system design (exoskeletons and end-effectors) and clinical research paradigms used, a consensus exists on the safety and value of robot-assisted UL therapy in reducing motor impairment, mainly at the shoulder and elbow (15, 33). The effects of robot-assisted therapy on muscle tone remains uncertain as only two reviews have specifically addressed this topic (34).

A recent systematic meta-analysis in 38 trials evaluated the effects of robot-assisted UL training in patients after a stroke on outcomes of motor control of the paretic upper limb, upper limb capacity, and basic ADL, in comparison with non-robotic treatment. Secondary outcomes were muscle strength and muscle tone. No serious adverse events were reported. Results reported significant improvements in UL motor control (about 2 points FMA UL sub-items) and muscle strength after robot-assisted training. Shoulder/elbow robotics showed small but significant effects on both motor control and muscle strength, while elbow/wrist robotics had small but significant effects only on motor control. Uncertain effects were reported for muscle tone assessed by the Modified Ashworth Scale (34). No effects were found for upper limb capacity and basic ADL. In the review by Bertani and colleagues 14 randomized controlled trials, two systematic reviews, and one meta-analysis were included (35). The Fugl-Meyer and Modified Ashworth scale were selected to measure primary outcomes, a measure of motor function and muscle tone, respectively. Functional independence measure and motor activity log were selected to measure secondary outcomes, such as activities of daily living. According to previous findings, the robot-assisted UL rehabilitation was more effective in improving upper limb motor function recovery, especially in chronic stroke patients than conventional therapy. No significant improvements were observed in the reduction of muscle tone or daily living activities (35).

Few studies have explored the combined effects of pharmacological treatments of UL spasticity and robot-assisted rehabilitation, so far (16, 36, 37). These preliminary results agree that greater improvements from the combined approach are expected in UL function, as assessed by the FMA. However, the training effects on spasticity and UL use in the ADL have found disagreement between the different studies (16, 36, 37).

With the limits of methodological differences among studies, the present study corroborates some elements of the existing literature (16). Some equivalence between the robot-assisted and conventional approaches was noticed in UL function, while no differences in the MAS were reported. Interestingly, the UL robot-assisted training increased UL muscle strength in specific muscles involved during functional tasks, outcome not included in earlier studies (16, 37). Historically, strengthening training has been a subject of controversy in stroke rehabilitation. However, this procedure is now included in post-stroke rehabilitation programs given the absence of adverse effects on spasticity and the positive consequences reported on strength and activity (3, 38). Strength training is commonly considered to be progressive resistance exercise. However, any intervention that involves attempted repetitive effortful muscle contractions can result in increased motor unit activity and increase strength (37). Besides, exercising entailing more numerous repetitions but with a reduced workload are recommended in post-stroke patients (31). There is growing evidence suggesting the crucial role of the treatment dose in functional recovery. However, a lack of consensus on the quantification of training intensity and its relationship with UL recovery patterns exists. Within this perspective, the ratio between the number of repetitions divided by the total time dedicated to the training has been reported to be a useful parameter to quantify training intensity and efficiency (31). These data could be relevant to evaluate the cost-effectiveness of technology-mediated rehabilitation as robot-assisted training. Robotics, in fact, allows implementing high-standardized training in term of numbers of repetition and progression of intensity and workload over time (15). The knowledge of the underlying neuromuscular mechanisms is of great interest in neurorehabilitation. With the limitations of the single-group analysis and the qualitative inspection of data, sEMG can reveal myographic activity reflecting the physiological processes that occur following cortical damage and changes promoted by rehabilitation training (39). The surface EMG assessment in this perspective allowed to observe specific impairments in proximal agonist muscle activity in the hand-to-mouth task, as reported in Figure 4 and as an example in two patients in Figure 5. Before treatment, the decrease of the biceps brachii, clavicular deltoid, and pectoralis major was higher than that observed in the healthy subjects suggesting an impairment in the eccentric contraction of elbow flexors and modulation of the internal shoulder rotator during the return (second) phase of the task. Five distinctive UL spasticity patterns has been identified for the position of the shoulder, elbow, forearm, and wrist joints (39). The most frequent (41.8%) is characterized by internal rotation and adduction of the shoulder and flexion at the elbow coupled with a neutral positioning of the forearm and wrist (40). The significance to recognize these patterns is essential to guide specific pharmacological and rehabilitative interventions (7, 39). The instrumental assessment of these patterns during UL movement using the sEMG analysis can reveal dynamic pattern of altered activation during specific task phases to further customize rehabilitation procedures. The results suggested that Armotion could act selectively on these proximal muscles. At the end of the treatment, the mean time of sustained activation of the biceps brachii was much longer if compared to the activation observed pre-treatment. The analysis of the mean sEMG envelope of single subjects is an example of how the robot-assisted training might improve the modulation of elbow flexors muscles (Figure 4, top left) and increase their activation (Figure 4 top right) during active task. Similarly, Pennati et al. performed a qualitative analysis of sEMG graphic records during two gestures: reaching the wall and return to the mouth and reaching movement to a visual target (16). Their results showed an improvement in muscle activation pattern and a reduction of co-contraction of agonist-antagonist muscles after the robotic exercises.

The strengths of the present study are the relatively large patient sample and the low drop-out rate, which suggest the feasibility of robotic training. The EMG analysis using a standardized experimental protocol to investigate the training effects on muscle activity are further strengths of this study, albeit only in a subgroup of patients (experimental group). The study limitations are the lack of a functional assessment after the treatment, measurement on activities of daily living and participation and patient related outcome. In addition, any patient stratification by the degree of impairment, the lack of follow-up assessment and neuroimaging support were reported.

This preliminary study has implications for practice and research. These findings suggest that UL robot-assisted strengthening interventions combined with pharmacological neurolysis increase strength and do not increase spasticity. Thus, strengthening programs should be part of rehabilitation after stroke to improve function and activity. Robot-assisted rehabilitation offers a wide range of training modalities that can be chosen for an individualized treatment in terms of assistance (passive, active-assisted, active) and perturbation. Research would be oriented toward ideal training models (i.e., number of repetitions, progression, duration) and how long the training effects last after the intervention. Surface EMG should be part of this multidisciplinary intervention to characterize specific pattern and to focus the training exercises to specific muscle impairment. It could help researchers to design studies with accurate patients' selection and stratification with specific impairments and similar likelihood of responding to rehabilitation. It is essential to draw up recommendations for a therapeutic guide of UL spasticity management in chronic stroke patients with UL spasticity.

To conclude, upper limb robot-assisted training holds promise when combined with botulinum toxin in improving upper limb strength and muscular activation pattern in patients with chronic stroke and spasticity.

## Author Contributions

MaG and NV have made substantial contributions to conception and design. MF and AP participated in the enrollment phase and carried out BoNT treatment procedures. ED and MiG carried out the clinical assessment. NV carried out the EMG assessments. SM and EB designed the algorithm for EMG data analysis. NV and ED participated in the manuscript draft and revision process. MaG and NV participated in the study design and coordination, and statistical analysis. MaG drafted the manuscripts and revision process. NS, LS, and AS participated in the manuscript revision and gave the final approval of the version.

### Conflict of Interest Statement

The authors declare that the research was conducted in the absence of any commercial or financial relationships that could be construed as a potential conflict of interest.
